# Sonication as a tool for disrupting biofilms and recovering microorganisms in bladder catheters

**DOI:** 10.1590/2175-8239-JBN-2022-0129en

**Published:** 2023-05-08

**Authors:** Juliette Cieslinski, Victoria Stadler Tasca Ribeiro, Camila Kowodzeichak de Lima, Letícia Kraft, Paula Hansen Suss, Felipe Francisco Tuon

**Affiliations:** 1 Pontifícia Universidade Católica do Paraná, Escola de Medicina, Laboratório de Doenças Infecciosas Emergentes, Curitiba, PR, Brazil.

**Keywords:** Bladder catheter, Microorganisms, Urinary, Biofilm, Sonication, Cateter vesical, Microrganismos, Urinário, Biofilme, Sonicação

## Abstract

**Introduction:**

Urinary catheter-related infection is commonly associated with bacterial biofilm. The impact of anaerobes is unknown, but their detection in the biofilm on this device has not been previously reported. This study aimed to evaluate the capability to recovery strict, facultative, and aerobic microorganisms in patients using bladder catheters from ICUs using conventional culture, sonication, urinary analysis, and mass spectrometry.

**Methods:**

Parallel, sonicated bladder catheters from 29 critically ill patients were compared with their routine urine culture. Identification was performed using matrix-assisted laser desorption/ionization with time-of-flight mass spectrometry.

**Results:**

The positivity rate in urine (n = 2, 3.4%) was lower than that in sonicated catheters (n = 7, 13.8%).

**Conclusion:**

Bladder catheter sonication showed more positive culture results than urine samples for anaerobic and aerobic microorganisms. The role of anaerobes in urinary tract infection and catheter biofilm is discussed.

## Resumo

**Introdução:**

A infecção relacionada ao cateter urinário é comumente associada ao biofilme bacteriano. O impacto dos anaeróbios é desconhecido, mas sua detecção no biofilme deste dispositivo não foi relatada anteriormente. Este estudo teve como objetivo avaliar a capacidade de recuperar microrganismos estritos, facultativos e aeróbios em pacientes que utilizam cateteres vesicais de UTIs utilizando cultura convencional, sonicação, análise urinária e espectrometria de massa.

**Métodos:**

Paralelamente, foram comparados cateteres vesicais sonicados de 29 pacientes gravemente enfermos com sua urocultura de rotina. A identificação foi realizada utilizando dessorção/ionização a laser assistida por matriz com espectrometria de massa por tempo de voo.

**Resultados:**

A taxa de positividade na urina (n = 2; 3,4%) foi inferior à dos cateteres sonicados (n = 7; 13,8%).

**Conclusão:**

A sonicação do cateter vesical apresentou resultados de cultura mais positivos do que as amostras de urina para microrganismos anaeróbios e aeróbios. É discutido o papel dos anaeróbios na infecção do trato urinário e no biofilme do cateter.

## Introduction

Urinary catheter use is an important risk factor for the development of urinary tract infections due to time-related bioburden. When an indwelling urinary catheter is inserted, it becomes colonized with microorganisms that can attach to the medical device, forming colonies that can be enclosed in a polymer matrix known as biofilms^1,2^. The biofilm can contain single or multiple species; the organisms involved can be anaerobic and/or aerobic bacteria and fungi, and many of these biofilms can induce serious complications^3,4^.

Various methods have been used to identify the bacterial population embedded in a biofilm. The microbiological evaluation of the biofilm can be done by qualitative, quantitative, and semi-quantitative techniques^5^. For quantitative analysis, gentian violet staining can be used, but it does not assess the presence of live bacterial cells, only the extracellular matrix of the biofilm. With this staining, it is possible to assess the presence or absence of a biofilm and quantify it through spectrophotometry after removal of the biofilm by sonication^6^. The plate rolling technique, similar to venous catheter tip culture, can also be used, being considered a semi-quantitative technique, where the probe tip is slid over a culture plate and then the cells can be counted. Techniques that remove the biofilm, such as sonication or vortexing, can be used for quantification, with sonication being a more appropriate method, as it has a better biofilm removal capacity^7^. Sonication is a method used to evaluate infection associated with invasive medical devices, as it allows removal of microorganism-associated biofilm^8^. Anaerobic bacteria (*Bifidobacterium* spp., *Bacteroides* spp., *Veillonella* spp., *Eubacterium* spp., *Anaerococcus* spp., *Prevotella* spp.) can be identified in 25% of urinary samples from patients in the intensive care units (ICUs). However, the role of these microorganisms in the initiation and perpetuation of urinary tract infection in this setting remains unclear^9^.

Unfortunately, most studies on the prevalence of anaerobes in the urine from critically ill patients with urinary catheter are outdated and use non-standardized methods of identification, such as matrix-assisted laser desorption/ionization with time-of-flight mass spectrometry (MALDI-TOF MS), which is the current gold standard for bacterial identification. Furthermore, these studies only examined urine, not the presence of the microorganism in the urinary catheter biofilm.

Considering these aspects and the scarce literature on microorganisms associated with urinary tract and bladder catheter biofilm, we evaluated the capability to recover strict, facultative, and aerobic microorganisms in bladder catheters of ICUs patients using conventional culture, sonication, urinary analysis, and mass spectrometry.

## Methods

**Figure 1.**
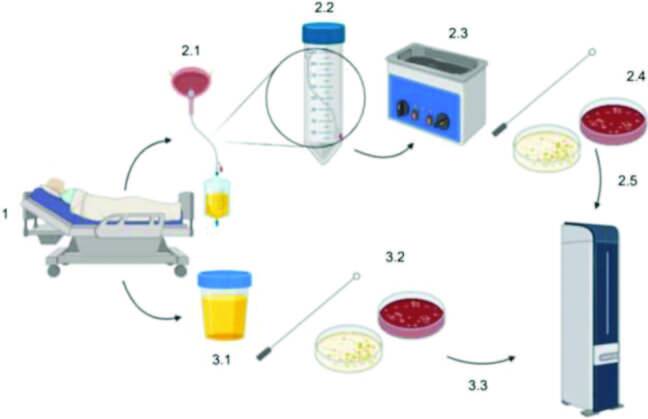
Recovery of samples (urine and bladder catheter) from 29 patients. 1: Patient in ICU was selected. 2.1: Recovery of bladder catheter. 2.2: Bladder catheter preparation. 2.3: Sonication. 2.4: Sonicated fluid cultivation. 2.5: After incubation, isolated colonies were identified by MALDI-TOF. 3.1: Urine recovery. 3.2: Urine was immediately subjected to microbial culturing. 3.3: After incubation, isolated colonies were identified by MALDI-TOF. Samples of both groups were cultured aiming to isolate aerobic and anaerobic microorganisms.

This was a retrospective study using samples of urine and bladder catheter from 29 patients admitted to the ICUs of Hospital Universitário Cajuru (Curitiba, Paraná, Brazil) between August and September 2018. After recovery, the urine was plated onto an anaerobic agar plate (Anaerinsol-S agar, Probac do Brasil, São Paulo, Brazil) for culturing strict anaerobic microorganisms and on a blood agar plate (Laborclin – A Solabia Group, Pinhais, Brazil) for culturing facultative anaerobic and aerobic microorganisms (for 72 and 48 h at 36°C, respectively). For sonication, the catheters were placed into a sterile 50-mL conical tube. Then, the tube was submerged in Ringer’s Lactate solution and vortexed for 30 s, followed by sonication using an ultrasonic bath (Sanders, Minas Gerais, Brazil) at 40 kHz at 37°C for 5 min and vortexed again for 30 s^10^. After this procedure, the sonicated liquid was plated onto Anaerinsol-S and blood agar for quantification (as described above). Figure 1 illustrates the samples recovering process flow. This study evaluated the prevalence of these microorganisms but not the association with confirmed urinary tract infection. Identification was performed using MALDI-TOF MS (Bruker Daltonik GmbH, Bremen, Germany). Continuous variables are reported as mean with standard deviation (±SD) or median and interquartile range, while categorical variables are reported as frequencies or percentages.

## Results and Discussion

Twenty-nine patients were included in this study, being ten women (34.5%) and 19 men (65.5%). Our study found a lower positivity rate in urine than in catheters for strict anaerobic microorganisms. Only 3.4% of urine samples showed anaerobic growth, whereas 13.8% of catheter samples were positive for strict anaerobic microorganisms on culture. For facultative anaerobic and aerobic microorganisms, only 41.4% of the urine samples showed aerobic growth, whereas 72.4% of the catheter samples were positive on culture.

MALDI-TOF was able to identify two anaerobic microorganisms in urine samples and seven in sonicated bladder catheter samples, as well as 13 aerobic microorganisms in urine samples and 25 in sonicated bladder catheter samples (Table 1). Agreement of positivity between samples was 100% with both methods. However, the positivity rate was higher in catheter samples than in urine samples. Only one patient’s urine and catheter tested positive on anaerobic culture (Table 2).

**Table 1.** Microorganisms (aerobic and anaerobic) in urine and bladder catheter identified by MALDI TOF

Sample	Type	Microorganism	n (%)
**Urine**	**Aerobic (n = 12)**	*Aspergillus fumigatus*	1 (8.3%)
		*Candida glabrata*	1 (8.3%)
		*Enterobacter cloacae*	1 (8.3%)
		*Enterococcus faecalis*	1 (8.3%)
		*Enterococcus faecium*	1 (8.3%)
		*Escherichia coli*	2 (16.7%)
		*Morganella morganii*	1 (8.3%)
		*Proteus mirabilis*	1 (8.3%)
		*Pseudomonas extremorienalis*	1 (8.3%)
		*Staphylococcus epidermidis*	1 (8.3%)
		*Staphylococcus capitis*	1 (8.3%)
	**Anaerobic (n = 2)**	*Peptostreptococcus anaerobius*	1 (50.0%)
		*Finegoldia magna*	1 (50.0%)
**Bladder catheter**	**Aerobic (n = 25)**	*Candida albicans*	1 (4.0%)
		*Candida glabrata*	1 (4.0%)
		*Corynebacterium striatum*	1 (4.0%)
		*Enterococcus faecium*	1 (4.0%)
		*Enterococcus faecalis*	1 (4.0%)
		*Enterobacter cloacae*	1 (4.0%)
		*Enterococcus faecalis*	6 (24.0%)
		*Escherichia coli*	4 (16.0%)
		*Morganella morganii*	1 (4.0%)
		*Pseudomonas aeruginosa*	2 (8.0%)
		*Proteus mirabilis*	1 (4.0%)
		Polimicrobial flora	1 (4.0%)
		*Staphylococcus epidermidis*	2 (8.0%)
		*Staphylococcus lugdunensis*	1 (4.0%)
		*Staphylococcus haemolyticus*	2 (8.0%)
	**Anaerobic (n = 7)**	*Peptoniphilus harei*	1 (14.3%)
		*Peptostreptococcus anaerobius*	1 (14.3%)
		*Petoniphilus assaccharolyticus*	1 (14.3%)
		*Prevotella bivia*	2 (28.6%)
		*Prevotella disiens*	2 (28.6%)

**Table 2.** Identification and quantification of leukocytosis in urine and bladder catheter samples with both aerobic and anaerobic growth

Patient ID	Gender	Urine Leukocytes	Aerobic urine CFU/mL	Aerobic urine MALDI TOF	Anaerobic urine CFU/mL	Anaerobic urine MALDI TOF	Aerobic bladder catheter CFU/mL	Aerobic bladder catheter MALDI TOF	Anaerobic bladder catheter CFU/mL	Anaerobic bladder catheter MALDI TOF
1	Male	Not analyzed	>100,000	*Escherichia coli*	>100,000	*Peptostreptococcus anaerobius/ Finegoldia magna*	10,000	*Escherichia coli*	>100,000	*Petoniphilus assaccharolyticus*
2	Male	3,000	Negative	Negative	Negative	Negative	520	*Corynebacterium striatum*	>100,000	*Prevotella disiens/Peptostreptococcus anaerobius/Prevotella bivia*
3	Male	>1,000,000	>100,000	*Enterobacter cloacae*	Negative	Negative	>100,000	*Enterobacter cloacae*	>100,000	*Prevotella disiens/Peptoniphilus harei*
24	Female	>1,000,000	>100,000	*Proteus mirabilis*	Negative	Negative	>100,000	*Proteus mirabilis*	>100,000	*Prevotella bivia*

Our results showed a higher positivity rate in catheter samples than urine samples for both anaerobic and aerobic microorganisms, while another study reported a much lower positivity rate in urine than in catheters using culture^11^. The gold standard for catheter-associated urinary tract infections diagnosis is quantitative culture; however, routine urine cultures do not support the growth of anaerobic bacteria^12^. The presence of anaerobes in urine has been described, although rarely in association with infection. In 15,250 urine specimens, less than 2% were anaerobes and none associated with infection. The most common anaerobe was *Lactobacillus*, followed by *Clostridium*, *Bacteroides*, *Peptostreptococcus,* and *Peptococcus*. These microorganisms are commonly found in regional microbiota (vaginal and intestinal), suggesting the possibility of contamination of the sites^13^.

The first study to identify anaerobes in patients with indwelling urethral catheters was published in 1976. In a study of 13 patients with long-standing indwelling catheters, anaerobes (*Bifidobacterium* sp, *Clostridium* sp, and *Veillonella* sp) were detected in urine obtained by percutaneous suprapubic needle aspiration to avoid contamination^6^. Anaerobic bacteria > 10^3^ per mL of urine were detected in > 5% of specimens obtained from suprapubic bladder aspirates, including *Peptostreptococcus*, *Veillonella, Bacteroides, Eubacterium*, *Clostridium*, and *Bifidobacterium* species^14^. However, 15% of anaerobes identified in urine specimens were antibody-coated, suggesting a potential role in urinary infection^15^.

This study had several limitations, including the fact that it included only 29 patients. We only examined patients with catheters who were hospitalized in the ICU and known to be at risk for bacteriuria and urinary tract infections. Thus, the results cannot be generalized to other populations. The real pathogenicity of these microorganisms was not evaluated, but the higher positivity of sonicated cultures suggests that these microorganisms are associated with biofilm. Biofilm is defined as a community of microorganisms adhered to a surface and surrounded by a self-created extracellular matrix. *Finegoldia magna* and *Prevotella* sp. have been found to adhere strongly to abiotic surfaces and develop as biofilms^16^. *Peptostreptococcus anaerobius* has also been associated with oral biofilm formation^17^; however, there is still no clarity on the biofilms formed by anaerobic microorganisms. Anaerobic microorganisms temporarily colonize the urinary tract, suggesting a potential role in urinary infection.

Bladder catheter sonication showed more positive culture results than urine samples for anaerobic and aerobic microorganisms. It is known that the use of a urinary catheter can increase the risk of developing bacteriuria by 3–7% for daily catheterization^18^. Moreover, strict anaerobic microorganisms may occur in the bladder catheter, and sonication can be an alternative way to dislodge and recover microorganisms from the material as it can detach biofilm and microorganisms from the surface. The results found in our study corroborate previous data and indicate that anaerobic bacteriuria is common in ICU patients with catheters^12^. The clinical significance of this study is related to the presence of anaerobes in biofilms, suggesting that these pathogens can be associated with infection and with biofilm formation. 

## Conclusion

Further studies are necessary to understand the pathogenicity and mechanisms of anaerobic bacteria and their role in infections of patients with catheters. We also hypothesized that these anaerobes can contribute to biofilm formation, increasing the complexity of the bacterial community as a symbiotic environment for pathogenic microorganisms. This study highlights the need to confirm the importance of anaerobic bacteria in the development and maintenance of biofilm and the need to treat or not treat these infections.
